# Case Report: Successful Treatment of Kaposi’s Sarcoma With Anlotinib in an HIV-Negative Patient After the Treatment of Drug Reaction With Eosinophilia and Systemic Symptoms Accessory Tragus

**DOI:** 10.3389/fmed.2022.907345

**Published:** 2022-05-25

**Authors:** Min Lin, Renwei Luo, Peng Zhang, Zhixun Xiao, Ting Gong, Chao Ji

**Affiliations:** ^1^Department of Dermatology, The First Affiliated Hospital of Fujian Medical University, Fuzhou, China; ^2^Central Laboratory, The First Affiliated Hospital of Fujian Medical University, Fuzhou, China

**Keywords:** Kaposi’s Sarcoma, anlotinib, tyrosine kinase inhibitors, HHV-8, DRESS

## Abstract

Kaposi’s Sarcoma (KS) is a neoplasm derived from endothelial cells and is associated with human herpesvirus-8 (HHV-8) infection. It is mostly seen in patients suffering from AIDS and/or chronic immunosuppression. Currently, systemic chemotherapy is the primary treatment option for the advanced KS. However, there is no consensus on the treatment of KS. In this case, an 84-year-old man with a history of psoriasis developed multiple painful dark purple nodules on the trunk and extremities during the treatment of drug reaction with eosinophilia and systemic symptoms (DRESS). KS was confirmed by the skin biopsy, and the immunohistochemical staining demonstrated the positivity for HHV-8 while the anti-HIV test was negative. The patient then received anlotinib treatment, a tyrosine kinase inhibitor, for 5 months, and his skin lesions subsided. This case indicates that anlotinib may be a potential treatment option for KS.

## Introduction

Kaposi’s Sarcoma (KS) is an HHV-8 associated angiogenic sarcoma that predominantly develops in lower extremities and was first described in 1872 by Kaposi (1). Drug reaction with eosinophilia and systemic symptoms (DRESS) is a severe adverse drug reaction characterized by fever, widespread rash, internal organ involvement, and eosinophilia. Several emerging therapies have been developed to treat most KS variants, including PD-1 inhibitor, multi-tyrosine kinase inhibitors, a novel kinase inhibitor, and IL(interleukin)-6 receptor inhibitor. Anlotinib is an oral novel multi-target tyrosine kinase inhibitor that achieves therapeutic efficacy in a variety of tumors, including lung cancer (2), advanced medullary thyroid cancer (3), soft tissue sarcoma, and metastatic renal cell carcinoma (4, 5).

## Case Report

**FIGURE 1:**
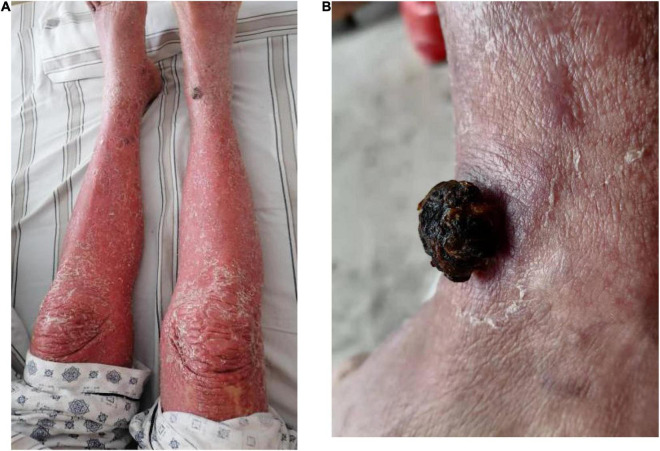
Clinical pictures of the patient. **(A)** One 2.0 × 2.5 cm purple papule on the right limbs after DRESS syndrome improved. **(B)** 2.5 × 2.5 cm purple papule on the right limbs after DRESS was cured.

**FIGURE 2:**
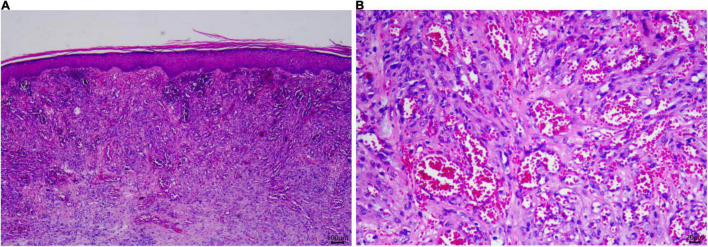
Skin histopathology of the patient. **(A)** A biopsy specimen of the right limb showed spindle cell proliferation, with intermingled erythrocytes seen in the dermis (Hematoxylin and eosin, ×25). **(B)** Significant atypia of endothelial cells. (Hematoxylin and eosin, ×100).

**FIGURE 3:**
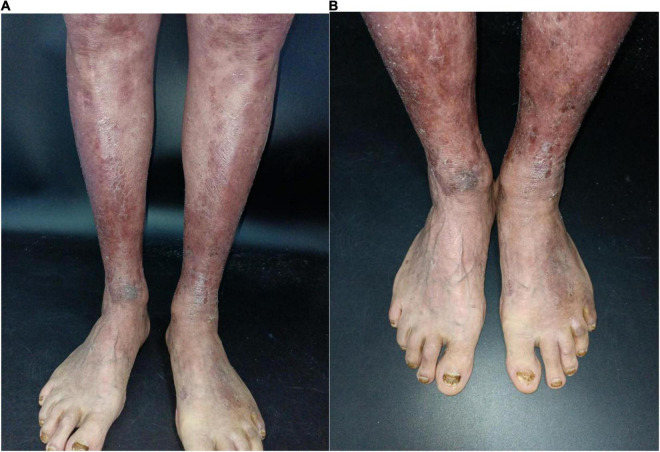
**(A,B)** Clinical pictures of the patient. After 4-month treatment with anlotinib, nodules begin to subside, leaving dark brown violaceous patches.

An 84-year-old man presented with multiple dark purple and painful nodules on the trunk and extremities. He had a history of psoriasis for 3 years and was well-controlled by acitretin and topical corticosteroids. Body surface area (BSA) of the initial psoriasis was 13%, and the psoriasis area and severity index (PASI) score was 17.6. About 9 months ago, the patient was admitted to the hospital 3 weeks after taking traditional Chinese medicine. He presented with fever, mild facial edema, widespread erythema (BSA = 75%), and icteric sclera. The patient’s laboratory results were as follows: Eosinophil count of 2,640/μl; alanine aminotransferase, 283 U/L; aspartate aminotransferase, 173 U/L; serum lipase(Rate method) of 163 u/L; and hemoglobin of 89 g/L. The patient was assessed using a diagnostic score according to the RegiSCAR. The final score was six, suggesting probable DRESS syndrome. Methylprednisolone (1 mg/kg/day) and plasmapheresis were immediately administered. After 1 month of admission, the DRESS syndrome improved, but two localized purplish nodules were noticed in his lower limbs. The nodules were painful with a coarse texture and had a diameter of 2–3 cm (Figures 1A,B). Then, the number of nodules were increasing in 1 month, and finally, the lesions appeared on the trunk and extremities, mostly in the lower extremities. No other abnormalities were noted on laboratory examination. Fecal occult blood test and anti-HIV test were negative. We initially suspected Kaposi’s sarcoma. In order to rule out the diagnosis of hemangioma and angiosarcoma, as well as to confirm the diagnosis, a skin biopsy was taken from the purple nodule over the right calf. Histopathological examination of the sections demonstrated abnormal vessels and spindle cell proliferation, with intermingled erythrocytes (Figures 2A,B). Immunohistochemical staining demonstrated positivity for HHV-8. PET-CT did not show visceral involvement. Given the patient’s anemia, pegylated liposomal doxorubicin (PLD) and paclitaxel were not recommended. After comprehensive consideration, the patient received anlotinib 8 mg/qod after being informed of the benefits and risks. The most common adverse reaction of anlotinib is hypertension. Other adverse reactions include hand-foot syndrome, hyperlipidemia, and gastrointestinal reactions. His blood pressure was slightly elevated (5–10 mmHg higher than usual) after taking anlotinib, but was still within the normal range. After 5 months of treatment, relief from pain was observed in patients and nodules began to subside, leaving dark brown, violaceous patches (Figures 3A,B). At present, the adverse effects caused by treatment were mild and tolerable, the patient is still in follow-up.

## Discussion

Kaposi’s sarcoma can be divided into four recognized clinical subtypes: sporadic or classical subtypes described by Kaposi, endemic disease subtypes observed in sub-Saharan Africa, epidemic subtypes observed in patients infected with human immunodeficiency virus (HIV), and iatrogenic subtypes observed in patients receiving immunosuppression, especially organ transplant recipients (6). DRESS is a potentially life-threatening, drug-induced hypersensitivity reaction that often demonstrates skin eruption and internal organ involvement. Kidney or liver failure is often observed in DRESS syndrome, which generally occurs with greater frequency in situations where chemically reactive metabolites have accumulated (7). On admission, disseminated KS was excluded based on clinical symptoms and PET-CT scan findings. KS presenting with local purple nodules in the lower extremities needs to be differentiated from bacillary angiomatosis, spindle cell hemangioma, and variants of fibrous histiocytoma (8). Due to the patient’s history of anemia, we recommended other options in addition to the first-line treatment. Finally, patients chose anlotinib for treatment after considering the drug’s side effects and their own economic situation. The patient was treated with 5 months of anlotinib. Then, disease progression is controlled and nodules are eliminated. In recent years, for local lesions in KS, several local therapies have been developed, including radiation therapy (9), intralesional chemotherapy (10), and electrochemotherapy (11). Topical treatments of dimiquimod or topical 9-*cis*-retinoid acid can also be used (12). PLD and paclitaxel are used as first-line agents for systemic therapy in the European guidelines in 2019 (6). But PLD and paclitaxel can induce myelosuppression, which limits the application (13, 14).

During KS formation, endothelial cells infected with HHV-8 induce vascular endothelial growth factor (VEGF) and its receptor (VEGFR) expression to participate in the activation of angiogenesis, thereby inducing the formation of new blood vessels (15). An increasing number of preclinical studies have shown that anti-angiogenic drugs are able to promote tumor vascular normalization. Bevacizumab, a promising targeted treatment, is the anti-VEGF monoclonal antibody. A phase II clinical trial showed that bevacizumab combined with PLD had a positive effect on patients with KS, with a PFS of 6.9 months and an ORR of 56% (16). Imatinib, a tyrosine kinase inhibitor, was found to be a potent inhibitor of Bcr-Abl, platelet-derived growth factor receptors(PDGFR), and the c-kit receptor. Imatinib has been shown to be active in AIDS-KS in phase II clinical trials by Henry B (17). In addition, two trials evaluating the novel kinase inhibitor EphB4-HAS for KS (NCT02799485, NCT03993106) are under development. Talimogene laherparepvec is being developed as a Phase 2 study (KAPVEC) (NCT04065152). As a novel small molecule multi-target tyrosinase inhibitor, anlotinib exerts anti-tumor effects by suppressing the metastasis, angiogenesis, and cell growth *via* targets VEGFR1, VEGFR3, VEGFR2/KDR, PDGFR-α, c-kit, and fibrogenic growth factor receptors(FGFR). It may inhibit more targets than other RTK inhibitors (4, 18). The platelet-derived growth factor(PDGF) and VEGF receptors play critical roles in KS development. The PDGFR is expressed in KS tumor specimens, the addition of PDGF to cultured KS cells induced the expression of VEGF (19). This potential role of PDGFR and c-kit in KS cell proliferation and induction of angiogenesis through VEGF makes inhibition of these receptors an attractive therapeutic target.

The underlying mechanism of action of anlotinib on KS is related to VEGFR and PDGFR pathways. However, its potential in the treatment of KS has not been reported. Therefore, anlotinib may be a new potential treatment for KS. With future studies and increased clinical experience, anlotinib is expected to be used for the treatment of other cancers.

## Data Availability Statement

The original contributions presented in the study are included in the article/Supplementary Material, further inquiries can be directed to the corresponding authors.

## Ethics Statement

The studies involving human participants were reviewed and approved by Ethics Committee of the First Affiliated Hospital of Fujian Medical University. The patients/participants provided their written informed consent to participate in this study. Written informed consent was obtained from the individual(s) for the publication of any potentially identifiable images or data included in this article.

## Author Contributions

ML, RL, and PZ conceptualized and designed the study and wrote the manuscript. TG and CJ critically revised the manuscript for important intellectual content. PZ and ZX collected clinical pictures and analyzed data. All authors read and approved the final manuscript.

## Conflict of Interest

The authors declare that the research was conducted in the absence of any commercial or financial relationships that could be construed as a potential conflict of interest.

## Publisher’s Note

All claims expressed in this article are solely those of the authors and do not necessarily represent those of their affiliated organizations, or those of the publisher, the editors and the reviewers. Any product that may be evaluated in this article, or claim that may be made by its manufacturer, is not guaranteed or endorsed by the publisher.

## Supplementary Material

Supplementary Figure 1 The data of therapeutic interventions.

Supplementary Figure 2 Timeline with relevant data from episode.
