# Supplementary figures and images for: Case Report: Successful Treatment of Kaposi’s Sarcoma With Anlotinib in an HIV-Negative Patient After the Treatment of Drug Reaction With Eosinophilia and Systemic Symptoms Accessory Tragus
Source: Front Med (Lausanne). 2022 May 25;9:907345. doi: 10.3389/fmed.2022.907345 (PMC9174420; doi:10.3389/fmed.2022.907345)

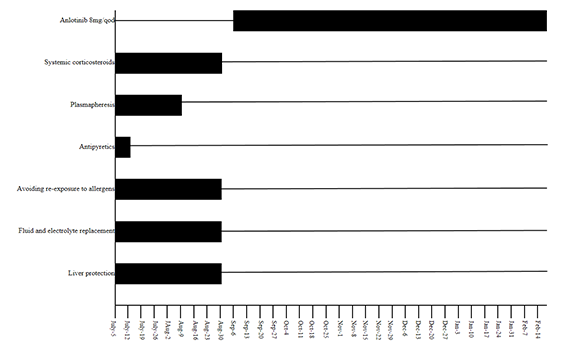

Supplement: Supplementary Figure 1 — The data of therapeutic interventions. [file Image_1.TIF]

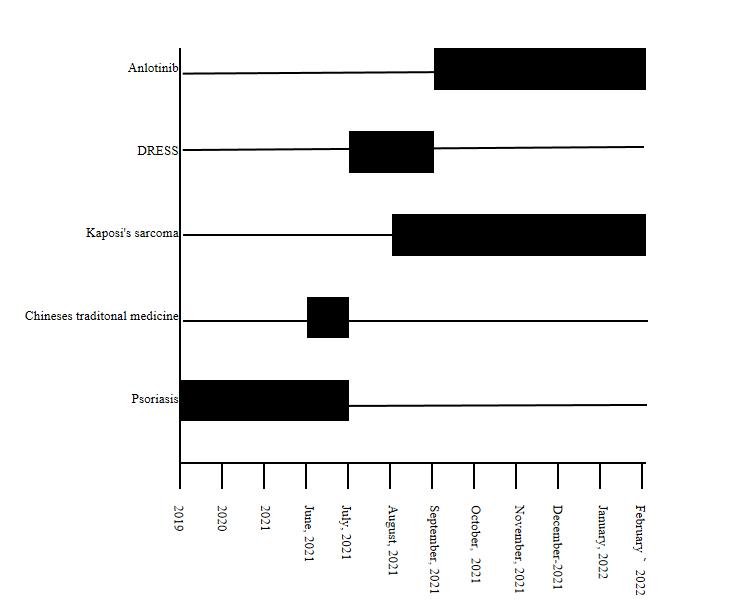

Supplement: Supplementary Figure 2 — Timeline with relevant data from episode. [file Image_2.JPEG]
